# Purification of an Exopolygalacturonase from *Penicillium viridicatum RFC3* Produced in Submerged Fermentation

**DOI:** 10.1155/2009/631942

**Published:** 2010-02-03

**Authors:** Eleni Gomes, Rodrigo Simões Ribeiro Leite, Roberto da Silva, Dênis Silva

**Affiliations:** Laboratory of Biochemistry and Applied Microbiology, Ibilce, Universidade Estadual Paulista (Unesp), Rua Cristovao Colombo, 2265, Jd. Nazareth, São José do Rio Preto, SP, CEP 15054-000, Brazil

## Abstract

An exo-PG obtained from *Penicillium viridicatum* in submerged fermentation was purified to homogeneity. The apparent molecular weight of the enzyme was 92 kDa, optimum pH and temperature for activity were pH 5 and 50–55°C. The exo-PG showed a profile of an exo-polygalacturonase, releasing galacturonic acid by hydrolysis of pectin with a high degree of esterification (D.E.). Ions Ca^2+^ enhanced the stability of enzyme and its activity by 30%. The *K*_*m*_ was 1.30 in absence of Ca^2+^ and 1.16 mg mL^−1^ in presence of this ion. In relation to the *V*_max_ the presence of this ion increased from 1.76 to 2.07 *μ*mol min^−1^mg^−1^.

## 1. Introduction

Pectinases are enzyme group that degrade pectic substances and are classified according to their mechanism of action in methylesterases (EC.3.1.11.1) that remove methoxyl groups from highly or partially esterified galacturonan; polygalacturonases catalyse the hydrolysis of the glycosidic bonds in a random fashion (endopolygalacturonase-EC.3.2.1.15) or from nonreducing end of homogalacturonan releasing galacturonic or digalacturunic acid residues (exopolyglacturonases EC.3.2.1.67 and EC 3.2.1.82) and lyases (PL) which cleave the glycosidic bonds by trans-elimination (pectato lyase-EC.4.2.2.2 and exopectato lyase-EC. 4.2.2.9) [1].

Production of pectinase by fungi generally is influenced by the composition of the medium, in particular the carbon and nitrogen sources and physicochemical conditions such as pH, temperature, and aeration, besides the fermentative system employed. Solid state (SSF) or submerged fermentations (SmF) have been proposed for enzyme production under laboratory conditions using agricultural and agroindustrial wastes, and several forms of PG have been purified from both fermentative processes [2, 3]. 

The purification of PG is an important tool for comprehension of its properties and reveals its structure and functional mechanism which are important to knowledge of the action of these enzymes in the plant infections process, in industrial application, and the importance of them in the biomass degradation. Fungal PGs generally are monomeric proteins with a carbohydrate content of 5–81% and molecular masses in a range from 13 to 82 kDa [4–8].

In a previous paper [9], we described the purification of an exo-PG produced by *Penicillium viridicatum* RFC3 in SSF, that enzyme exhibited a molecular weight of 24 kDa and was strongly stimulated by Ba^2+^. In this paper we report the production of polygalacturonase by the same fungus in SmF, with orange bagasse and wheat bran as carbon sources, and the purification of another exo-PG with different physicochemical properties from the earlier enzyme.

## 2. Material and Methods

### 2.1. Microorganism

The microorganism used was *Penicillium viridicatum* RFC3, isolated from decaying vegetables in São José do Rio Preto, SP, Brazil and maintained as stock culture on Sabouraud dextrose agar (Oxoid) containing 0.3% citrus pectin at 7°C.

### 2.2. Media, Cultivation of Microorganism, and Enzyme Production

Polygalacturonase was produced by submerged fermentation (SmF) in a 500 mL Erlenmeyer flask containing 100 mL of sterilized (120°C/30 minutes) liquid medium containing 3% carbon source (1.5% ground orange bagasse, provided by Bascitrus (Mirassol, SP) and 1.5% wheat bran) and 1 g L^−1^ of (NH_4_)_2_SO_4_ and MgSO_4_.7H_2_O. The medium was sterilized at 120°C for 30 minutes, inoculated with a volume of conidial suspension in 0.1% Tween 80 equivalent to 10^7^ spores per mL in the final medium and cultured at 28°C for 4 days. Every day, one flask was removed and the fermented material was filtered and centrifuged at 10,000 g for 15 minutes at 4°C. The supernatant was used as crude enzyme solution.

### 2.3. Enzyme Activity Measurements

Exopolygalacturonase (exo-PG) activity was assayed in a reaction mixture containing 1% citrus pectin solution with a degree of esterification (D.E.) of 92% (Sigma) in 0.2 M sodium acetate buffer (pH 4.5) at 45°C for 10 minutes. The number of reducing groups, released by enzymatic action, was measured by the DNS method [10] and expressed as galacturonic acid. One unit of enzyme activity (U) was defined as the amount of enzyme releasing one *μ*mol of galacturonic acid per minute under the assay conditions [3]. 

Endo-polygalacturonase (endo-PG) was measured viscosimetrically by adding 2.0 mL of crude enzyme to 6.0 mL of 0.2 M citrate-NaOH buffer (pH 5.5) containing 3% citrus pectin with 92% D.E. (Sigma). The reaction mixture was incubated at 45°C for 10 minutes, after which its viscosity was determined with a Basic viscosimeter (Fungilab). The blank contained thermally inactivated crude enzyme. One unit of enzyme activity (U) was defined as the amount of enzyme that reduced the initial viscosity by 50% per minute under assay conditions.

### 2.4. Enzyme Purification Procedure

A 1300 mL volume of crude enzyme solution obtained after 96 hours of fermentation was dialyzed overnight against 20 mM acetate buffer pH 4.5 in acetate cellulose membrane (Pharmacia) and concentrated by ultrafiltration with Quixstand Benchtop of GE Healthcare with a 10 kDa cut-off. The retentate was directly loaded on a Sephadex G-75 (Pharmacia) column (2.6 × 90 cm) equilibrated with 40 mM acetate buffer (pH 5.0) and eluted with the same buffer at a flow rate of 0.3 mL min ^−1^. Fractions of 4 mL were collected and assayed for PG activity. The objective of this procedure was to estimate the number of isoforms of PG present in the crude enzyme solution. 

In another assay, the same volume of crude enzyme was concentrated by ultrafiltration with Quixstand Benchtop of GE Healthcare with a 10 kDa cut-off and then filtered with a 50 kDa cut-off membrane and loaded directly on a Sephadex G-75 column following the procedure described above. 

Protein fractions collected from the Sephadex column, corresponding to the PG activity peak, were pooled and loaded on a Q Sepharose column (Aldrich 30 × 1 cm) equilibrated with 40 mM acetate buffer, pH 4.5. The adsorbed material was eluted with a linear gradient (0.0 to 1.0 M) of NaCl, in the same buffer, at a flow rate of 0.6 mL min ^−1^. The protein fraction with PG activity was desalted overnight by dialysis against 20 mM acetate buffer, pH 4.5, at 4°C.

### 2.5. Analytical Electrophoresis

The molecular weight of the purified enzyme was determined by SDS-PAGE in a Mini Protean II apparatus (10 × 8 cm) (Biorad). Electrophoresis was carried out on a 4% (w/v) polyacrylamide stacking gel and 10% (w/v) resolving gel in Tris/glycine buffer (pH 8.3) [11] with the Sigma molecular weight marker M6539 (6.5–180 kDa) in a parallel lane. The protein band was visualized by silver staining.

Analytical isoelectric focusing PAGE was performed in Ettan IPGphor II Isoelectric focusing system (Amersham) by electrophoresis in a 12.5% polyacrylamide gel (14 × 15 cm) containing 5% Pharmalyte (GE Healthcare), which established a pH gradient from pH 3.0–10.0, in accordance with the instructions of the supplier. The gel was silver-stained for protein determination.

### 2.6. Protein Estimation

Protein concentration was determined in the concentration ranges of 1–10 and 10–100 *μ*g mL^−1^ by the Bradford microassay [12], with bovine serum albumin (BSA) as the standard.

### 2.7. Properties of Purified Enzyme

All enzyme catalytic properties were assayed with 1% citrus pectin (D.E 92%, Sigma) as substrate using the procedure for enzyme activity determination described above and carried out with three replicates. PG activity was assayed as a function of pH, in McIlvaine buffer (pH 4.0–8.0), at 55°C, and temperature, in McIlvaine at the pH optimum, incubated at temperatures between 35°C and 70°C.

The thermal stability was investigated by remeasuring the activity of the purified enzyme solution after it had been kept for 1 hour, in the absence of substrate, at temperatures between 5 and 80°C. The half-life was determined by incubating the enzyme solution at 60°C for 1 hour. In these tests, the initial and final PG activities were determined at optimum pH and temperature.

pH stability of the purified enzyme was evaluated by dispersing (1 : 1, v/v) enzyme solution in 0.1 M McIlvaine buffer (pH 3.0–8.0) and 0.1 M glycine-NaOH buffer (pH 8.0–10.5) and maintaining these solutions at 25°C for 24 hours. An aliquot was taken to determine the remaining activity at the optimum pH and temperature.

The Michaelis constant (*K*_*m*_) and *V*_max_ values were determined from Lineweaver-Burk plots of enzyme activity measured with 92% D.E. citrus pectin (Sigma) as substrate, at concentrations between 1.0 and 10.0 mg mL^−1^ at optimum pH and temperature. The results were plotted with the program Grafit 5.0.

The effects of metal ions (Ag^+^, K^+^, Na^+^, Cu^2+^, Ca^2+^, Ba^2+^, Co^2+^, Hg^2+^, Ni^2+^, Mg^2+^, Mn^2+^, Zn^2+^, Cr^3+^, Al^3+^, Fe^3+^) and EDTA on enzyme activities were assayed at concentrations of 2.0, 5.0, and 10.0 mM in the reaction mixture.

The substrate specificity was assessed by testing polygalacturonic acid, citrus pectin (Sigma) of 26% and 92% D.E., and apple pectin (87% D.E.-Sigma) as substrates under optimum conditions for enzyme activity. 

The products of the hydrolysis of citrus pectin (92% D.E.) by PG were analyzed by paper chromatography on Whatman no. 1 paper, with ethyl acetate/isopropanol/water (6 : 3 : 1, by volume) as the mobile phase.

## 3. Results and Discussion

### 3.1. Production of PG by SmF

**Figure 1:**
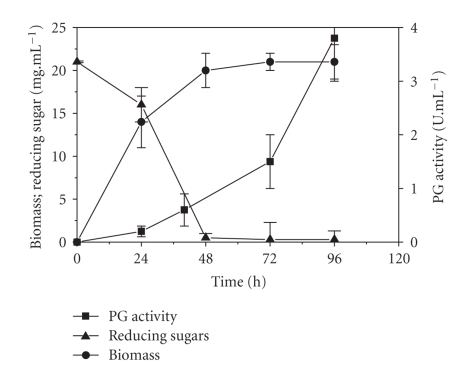
Production of PG by *P. viridicatum *RFC3 in submerged fermentation with orange bagasse (1.5%) and wheat bran (1.5%) as carbon sources.

During thesubmerged fermentation in medium containing orange bagasse and wheat bran as carbon sources (Figure 1), the PG activityincreased continuously between 24 and 96 hours of batch culture varying from 3.0 to 4.1 U mL^−1^ by three repetitions. The enzyme activity was lower than that obtained in SSF with the same fungus (18 U mL^−1^) [9]. Orange bagasse (pressed peel, pulp, rag, and seeds) has an average pectin content of 223 g Kg^−1^ (dry weight) [13, 14] and is a good inducer of pectinases [15, 16] while wheat bran is a good nutrient source [17]. In spite of this, the PG activity obtained was lower than that reported for enzyme production in SmF on other carbon sources. Patil and Dayanand [18] obtained 30.3 U mL^−1^ of exo-PG from the *Aspergillus niger* DMF 27 when they used deseeded sunflower and glucose as carbon sources. The same fungi produced 14.5 U mL^−1^ of pectinase in medium containing citrus pectin [19]. The related *A. sojae* produced 15.5 U mL^−1^ of pectinases in medium supplemented with maltrin [20].

PG production increased substantially when the reducing sugar content dropped and after the log phase of fungal growth. The consumption of reducing sugars promotes fungal growth and ensures success in colonization of medium [21]. On the other hand, a high level of reducing sugars in the medium is an important factor limiting of enzyme production, owing to catabolic repression [2].

### 3.2. Purification of PG

**Figure 2:**
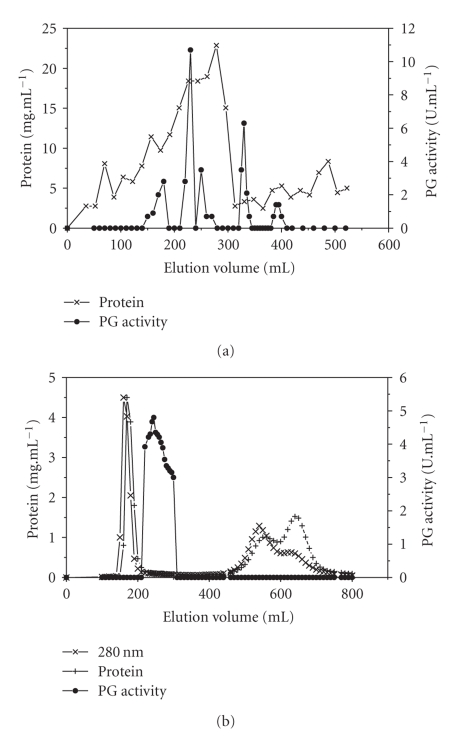
Gel filtration chromatography of PG on Sephadex G-75 (2.5 × 90 cm) equilibrated with 40 mM acetate buffer, pH 4. (a) Crude extract concentrated by ultrafiltration with 10 kDa cut-off, (b) crude extract after ultrafiltration with 10 and 50 kDa cut-off.

The crude enzyme solution obtained after 96 hours of fermentation, concentrated by ultrafiltration with a 10 kDa cut-off and separated on a Sephadex G-75 column, afforded 5 peaks of PG activity suggesting a number of isoforms (Figure 2(a)). In work published previously, we obtained 5 PG fractions by solid-state fermentation of *P. viridicatum* RFC3 on a 1 : 1 mixture (w/w) of wheat bran and orange bagasse at 67% moisture [17], while and recently in another experiment, 6 PG fractions were obtained in the same medium, but at 80% of moisture [9]. We supposed that the moisture difference between the two media could have influenced the expression of PG isoforms, but now, in liquid medium, we found the same number of isoforms as in the medium with lower moisture content. The influence of water potential (*a*_*w*_) variation on the isoform expression of extracellular enzymes has been attributed to changes in the permeability of fungal membrane, limitation of sugar transport, and presence or absence of inducer [22]. We have also reported changes in the expression of extracellular enzymes with variation of culture conditions and fermentative techniques [17].

The sequential productions of pectinases have been reported by other authors in various microorganisms [15]. Pectinases produced by the same microorganism exhibit differing molecular weights, degrees of glycosylation and specificities, due either to posttranslational modification of a protein from a single gene or to the expression of different genes, and such variants are important in balancing specific modes of action (endo or exo) and substrate affinity [5, 23]. 

**Figure 3:**
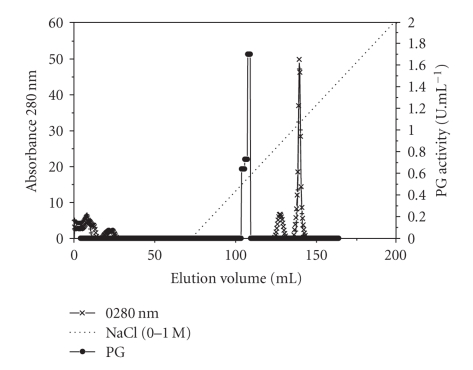
Ion-exchange chromatography of PG on Q Sepharose (1 × 30 cm) equilibrated with 40 mM acetate buffer, at pH 4.0 and eluted with NaCl (0–1 M).

**Figure 4:**
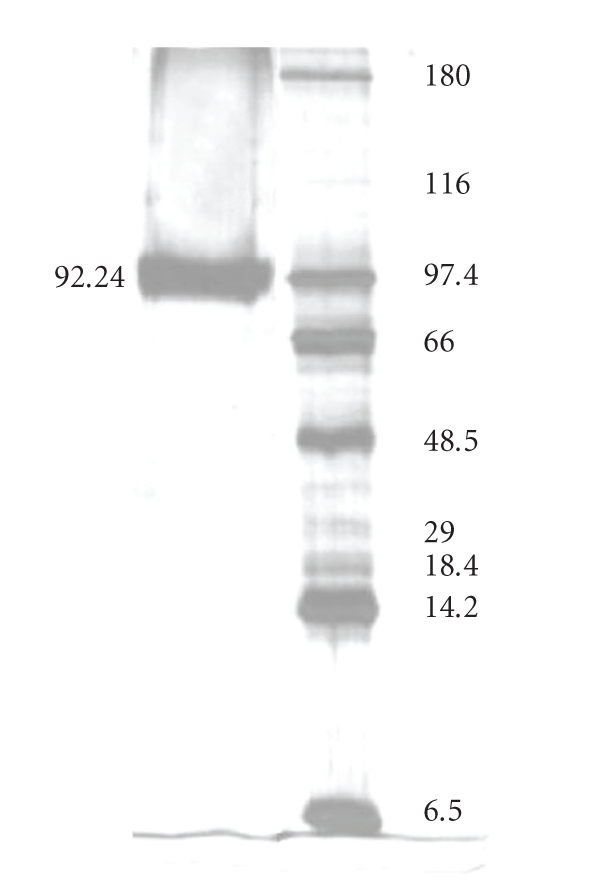
SDS-PAGE of purified PG.

When the crude enzyme solution was concentrated by ultrafiltration at 10 kDa and shortly after filtered through a 50 kDa cut-off membrane, and the concentrated material loaded on a Sephadex G-75 column, only one polygalacturonase peak (PG III) was eluted, indicating that the majority of the PG fractions from crude enzyme were of low molecular weight and only one (PG III) was retained by the 50 kDa membrane (Figure 2(b)). The PG peak, corresponding to elution volume 215 to 315 mL, was applied to a Q Sepharose column at pH 4 and, after elution with NaCl solution (around 0.5 M), only one peak of PG was detected (Figure 3). SDS/PAGE revealed that this PG peak was homogeneous (Figure 4). Three stages were necessary to reach a 37.7-fold PG III purification, with a final yield of 3.4% (Table 1).

### 3.3. Characterization of PG

Molar mass of PGIII was estimated to be 92.2 kDa (Figure 4). Homogeneity was confirmed by isoelectric focusing, where a single band with isoelectric point (p*I*) of 5.4 was observed (figure not shown).

**Figure 5:**
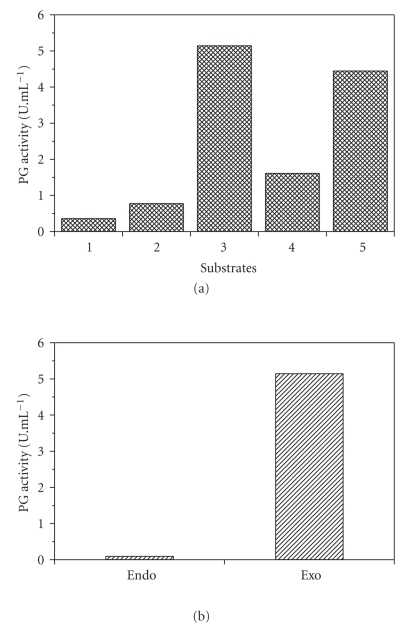
Properties of exo-PG III. (a) Substrate specificity: 1: polygalacturonic acid; 2: citrus pectin (26% D.E.); 3: citrus pectin (92% D.E.); 4: apple pectin (82 D.E.); 5: commercial citrus pectin (87% D.E.); (b) mode of action of PG (endo- and exo-activity).

Purified PG III exhibited higher activity on highly esterified pectin (Figure 5(a)) suggesting that PG is a polymethylgalacturonase. However, the activity on citrus pectin (5.1 U mL^−1^) was appreciably higher than that on apple pectin, although both have high D.E. (92% and 82%, resp.).

**Figure 6:**
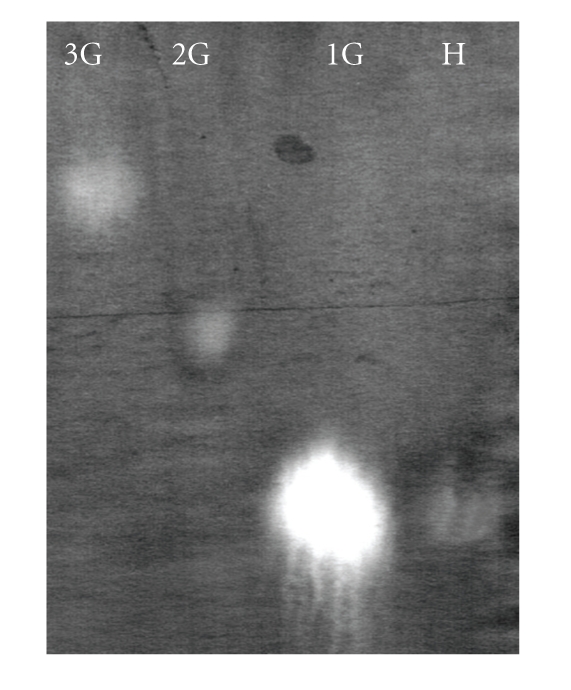
Products from hydrolysis of citrus pectin (92% D.E.) by exo-PG III—3G: trigalacturonic acid, 2G: digalacturonic acid, 1G: galacturonic acid, H: enzyme hydrolyzate.

The mode of action of the polygalacturonase on citrus pectin (92% D.E.) was also assessed by measuring viscosity in the reaction mixture, to estimate endo activity. Figure 5(b) shows that the enzyme had a poor capacity to decrease the viscosity of the pectin solution, indicating mainly exo-activity. Analysis by paper chromatography revealed that galacturonic acid was the sole product of enzyme hydrolysis after 5 minutes of incubation (Figure 6), reinforcing the evidence for an exopolygalacturonase (exo-PG). 

The effect of ions on exo-PG III activity was tested at concentrations of 2.0, 5.0, and 10.0 mM. The ions Hg^2+^, Zn^2+^, Mg^2+^, Fe^3+^ and Cu^2+^ strongly inhibited the exo-PG activity while, Cr^3+^, and K^+^ only partially inhibited it. On the other hand, ions such as Na^+^, Mn^2+^, and Ca^2+^, at 5 and 2 mM, enhanced the enzymatic activity by 10% (and Ca^2+^ by 30% at 5 mM). EDTA inhibited the enzyme activity by 7–17%, depending on the concentration (Table 2).

Na^+^ has been described as an inducer of PG activity by Chun and Huber [24]. Divalent ions such as Ca^2+^ act directly on the pectin molecule, stabilizing the negatively charged carboxyl groups and indirectly stimulating the polygacturonase activity [25, 26].

The concentration of the ions Cr^3+^, Al^3+^, Ag^+^, K^+^, and Ni^2+^ in the reaction mixture was related anomalously to their inhibitory effects, which were highest at the lowest concentration.

**Figure 7:**
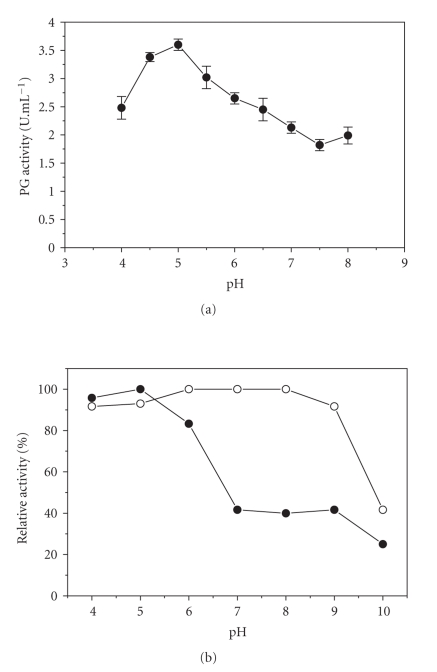
Properties of exo-PG III. (a) Effect of pH on enzyme activity; (b) effect of pH on enzyme stability. 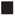: PG alone ◯: PG plus Ca^2+^.

Exo-PG III showed maximum activity at pH 5.0 and 50% of its maximum activity at pH 8.0 (Figure 7(a)). It was stable in the pH range 4–5, but the presence of Ca^2+^ ions in the reaction mixture resulted in an increase in the enzyme stability, with 90–100% of the full activity in a broader pH range of 4.0–9.0 (Figure 7(b)). 

**Figure 8:**
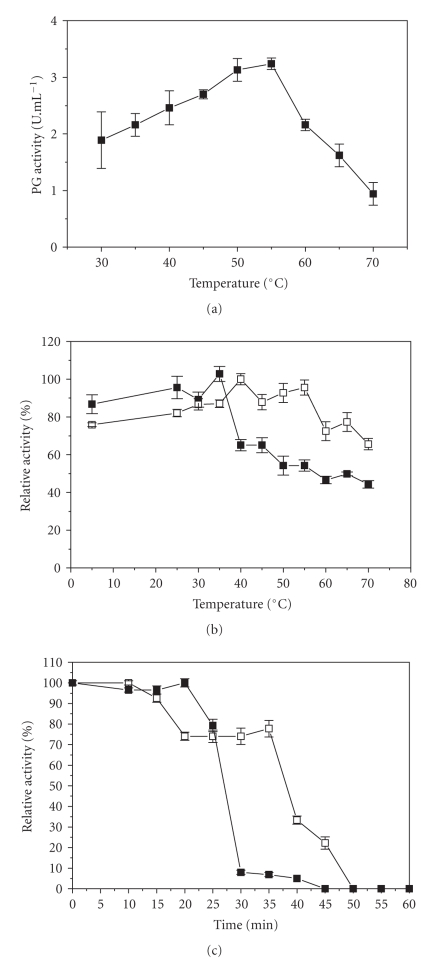
Properties of exo-PGIII. (a) Effect of temperature on enzyme activity; (b) effect of temperature on enzyme stability. (c) Enzyme stability at 60°C (half-life). ■: PG alone; □: PG plus Ca^2+^.

With respect to temperature, optimal PG activity was observed at 50–55°C (Figure 8(a)). In the absence of substrate for 1 hour, exo-PG III showed 85–100% of the original activity at 5–35°C, while at 70°C, the enzyme lost 55% of its initial activity. However, in the presence of Ca^2+^ (10 mM), the PG maintained 80–100% of the initial activity at 5–55°C and around 70% when maintained at 70°C (Figure 8(b)). In another experiment, exo-PG III was maintained at 60°C for 1 hour (samples taken every 5 minutes) in the presence and absence of Ca*^2+^*. Without this ion, the enzyme preserved 50% of its initial activity for 25 minutes, while in its presence, the enzyme half-life increased to 37 minutes (Figure 8(c)). According to Hernández et al. [27], Ca*^2+^* protects enzymes against thermal denaturation and plays a vital part in maintaining their active configuration at high temperatures.

The half-life time at 60°C of exo-PG III from *P. viridicatum *was higher than that described by Shanley et al. [28] for the three polygalacturonases purified from *P. pinophilum*. Those half-lifes were 10.6 minutes for PGI, 16.5 minutes for PGII, and 9.5 minutes for PGIII. However, the PG from *Acrophialophora nainiana* purified by Celestino et al. [29] had a half-life of 20 minutes at 60°C and 3 minutes at 70°C.

The exo-PG III affinity for citrus pectin (92% D.E.) was determined by the Lineweaver-Burk plot. In the presence of Ca^2+^, the substrate affinity increased, with *K*_*m*_ falling from 1.30 (±0.04) to 1.16 (±0.05) mg mL^−1^. Similarly, an improvement was observed in *V*_max_ in the in presence of this ion, with values rising from 1.76 (±0.06) to 2.07 (±0.03) *μ*mol min ^−1^ mg^−1^. 

Other purified exo-PGs have shown widely differing values in their kinetic parameters, which range from 0.11 mg mL^−1^ to 4.47 mg mL^−1^ for *K*_*m*_ and 1.68 *μ*mol min ^−1^ mg^−1^ to 1100 *μ*mol min ^−1^ mg^−1^ for *V*_max_ [30–33]. 

The properties of exo-PG III from *P. viridicatum* described in this work revealed quite different properties from those of another PG (exo-PG II) from the same fungus [9] such as molecular weight (92 and 24 kDa, resp.) and optimum pH of activity (5.0 and 6.0, resp.). Besides, the PG III, purified in this work, proved to be an exo-PG inhibited by to Ba^2+^ at 10 mM which enhanced the stability of exo-PG II, on the other hand, its stability against pH variation, thermostability, and substrate affinity were improved in presence of Ca^2+^.

The effect of Ca^2+^ and Ba^2+^ on stability and activity of exo-PGs brings out one step closer for improvement of pectinase efficiency when used in bioprocess application as juice extraction.

## Figures and Tables

**Table 1:** Summary of purification of exo-PG III from *Penicillium viridicatum* RFC3.

Step	Volume	PG	Protein	U total	S.A.*	Yield	Fold
	(mL)	(U mL^−1^)	(mg mL^−1^)		(U mg^−1^)	(%)	purification
Crude enzyme	1300	3.8	0.08	4966	49.0	100	—
Ultrafiltration	45	63.9	0.72	2875.5.4	88.8	57.9	9.6
in 10 and 50							
kDa cut-off							
membranes							
Fraction I from	198	5.1	0.03	1003.9	195.0	20.2	21.2
Sephadex G75							
column							
Fraction I from	35	4.9	0.014	169.8	346.4	3.4	37.7
Q Sepharose							
column.							

*S.A. (Specific Activity).

**Table 2:** Effect of metal ions on the exo-PG III activity.

Ions	Residual activity (%)					

	Ion concentration (mM)					
	2	S	5	s	*10*	
Control	100		100		100	
Al^3+^	67	±3.0	90	±10	99	±4
Fe^3+^	43	±5	15	±2	ND	
Cr^3+^	60	±5	83	±4	93	±5
Zn^2+^	3		0		0	
Hg^2+^	0		0		0	
Mn^2+^	110	±2	93	±3	90	±4
Mg^2+^	27	±2	ND		ND	
Cu^2+^	33	±4	ND		ND	
Ca^2+^	110	±2	130	±3	107	±1
Co^2+^	107	±3	100	±5	93	±2
Ag^+^	87	±3	90	±1	93	±1
Na^+^	123	±3	107	±2	98	±2
K^+^	63	±4	77	±5	84	±3
Ba^2+^	90	±1	90	±2	20	±1
Ni^2+^	63	±3	78	±2	81	±1
EDTA	93	±2	83	±3	83	±3

ND: not determined.

Data are mean of three repetitions.
